# Post-gastric endoscopic submucosal dissection suturing by the reopenable clip over line method using the clip with line pulley securing technique

**DOI:** 10.1055/a-2541-0042

**Published:** 2025-03-03

**Authors:** Hiroki Kato, Makoto Kobayashi, Hirotaka Takeshima, Hiroshi Nakayabu, Akihiro Maruyama, Shintaro Tominaga, Hitoshi Sugiyama

**Affiliations:** 1 37036 Gastroenterology, Yokkaichi Municipal Hospital, Yokkaichi, Japan

Bleeding post-endoscopic submucosal dissection (ESD) is a significant challenge. Various techniques have been developed to reduce the risk of post-ESD bleeding, one of which is the reopenable clip over line method (ROLM) reported by Nomura and colleagues, which is designed specifically to prevent hemorrhage following ESD procedures 1 . Although a final thread stopper is not always necessary in ROLM, securing the thread may enhance the stability of the suture. The locking-clip technique described by this groups achieves both thread closure and cutting simultaneously 2 , but it requires a certain level of familiarity to ensure a successful outcome.

In contrast, we employed the clip with line pulley securing (CLiPS) method reported by Ohata et al. to secure the threads in ROLM using an indwelling snare 3 . We performed ROLM suturing in six gastric ESD patients, with one of these patients continuing antithrombotic medication (edoxaban) during the procedure. For ROLM, we use a 3–0 nylon thread and a SureClip (Micro-Tech) with a 16-mm aperture. After completing the clipping, the end of the thread is passed through a disposable snare (Olympus ligature), which has been pretightened to expose only the loop tip before insertion into the forceps channel. The thread is then secured by tightening the snare and drawing it into the loop stopper. Finally, the thread and any excess portion of the snare are cut using a hook cutter M (ZEON Medical) ( Fig. 1 ; Video 1 ).

**Fig. 1:**
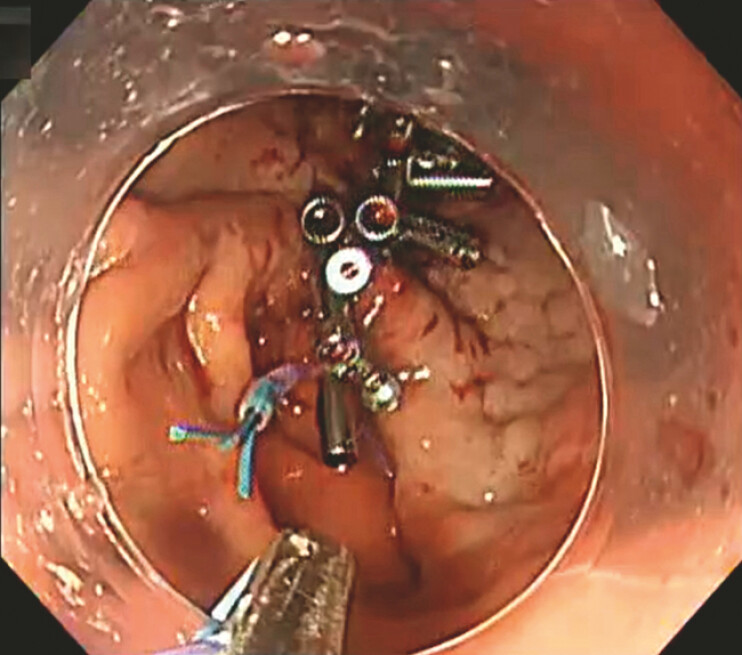
Endoscopic image showing the final appearance after suturing by the reopenable clip over line method (ROLM) and using the clip with line pulley securing (CLiPS) technique to fix the threads.

A disposable snare is used to lock the suture threads after reopenable clip over line method (ROLM) suturing has been performed following gastric endoscopic submucosal dissection.Video 1

The mean diameter of the resected specimens was 33.3 mm. The mean suture time was 20.3 minutes. The average number of clips used was 16.8. The average time required for thread closure was 43.6 minutes. This method allowed successful thread closure in all cases. No post-procedural hemorrhage or perforation occurred.

The method using the CLiPS technique is useful for fixing ROLM threads after gastric ESD.

Endoscopy_UCTN_Code_TTT_1AO_2AO

## Notes

Endoscopy E-Videos https://eref.thieme.de/e-videos
*E-Videos* is an open access online section of the journal *Endoscopy* , reporting on interesting cases and new techniques in gastroenterological endoscopy. All papers include a high-quality video and are published with a Creative Commons CC-BY license. Endoscopy E-Videos qualify for HINARI discounts and waivers and eligibility is automatically checked during the submission process. We grant 100% waivers to articles whose corresponding authors are based in Group A countries and 50% waivers to those who are based in Group B countries as classified by Research4Life (see: https://www.research4life.org/access/eligibility/ ). This section has its own submission website at https://mc.manuscriptcentral.com/e-videos .
